# Alterations of individual thalamic nuclei volumes in patients with migraine

**DOI:** 10.1186/s10194-019-1063-3

**Published:** 2019-12-09

**Authors:** Kyong Jin Shin, Ho-Joon Lee, Kang Min Park

**Affiliations:** 1 Department of Neurology, Haeundae Paik Hospital, Inje University College of Medicine, Haeundae-ro 875, Haeundae-gu, Busan, South Korea; 2 Department of Radiology, Haeundae Paik Hospital, Inje University College of Medicine, Busan, South Korea

**Keywords:** Migraine, Thalamus, Volume, Network

## Abstract

**Background:**

The aim of this study is to investigate the alterations of thalamic nuclei volumes and the intrinsic thalamic network in patients with migraine.

**Methods:**

We enrolled 35 patients with migraine without aura and 40 healthy controls. All subjects underwent three-dimensional T1-weighted imaging. The thalamic nuclei were segmented using the FreeSurfer program. We investigated volume changes of individual thalamic nuclei and analyzed the alterations of the intrinsic thalamic network based on volumes in the patients with migraine.

**Results:**

Right and left thalamic volumes as a whole were not different between the patients with migraine and healthy controls. However, we found that right anteroventral and right and left medial geniculate nuclei volumes were significantly increased (0.00985% vs. 0.00864%, *p* = 0.0002; 0.00929% vs. 0.00823%, *p* = 0.0005; 0.00939% vs. 0.00769%, *p* < 0.0001; respectively) whereas right and left parafascicular nuclei volumes were decreased in the patients with migraine (0.00359% vs. 0.00435%, *p* < 0.0001; 0.00360% vs. 0.00438%, *p* < 0.0001; respectively) compared with healthy controls. The network measures of the intrinsic thalamic network were not different between the groups.

**Conclusions:**

We found significant alterations of thalamic nuclei volumes in patients with migraine compared with healthy controls. These findings might contribute to the underlying pathogenesis of the migraine.

**Trial registration:**

None.

## Background

Migraine is a common disabling headache disorder, affecting between 10% and 20% of the population worldwide, [1] and is characterized by recurrent headaches of moderate to severe intensity, pulsating quality, and unilateral location. Headaches are aggravated by routine physical activity and are associated with nausea, photophobia, and/or phonophobia [2].

In recent years, neuroimaging technology has provided more convenient methods for better understanding the pathogenesis of neurological disorders. Several brain magnetic resonance imaging (MRI) studies have identified structural and functional changes in patients with migraine and have suggested that the alterations of these changes may be associated with the pathophysiology of migraine [3–7]. These changes have been demonstrated in patients with migraine in different phases of the disease [8]. In the prodrome period, hypothalamus, pons, spinal trigeminal nuclues and visual cortex may be involved, and viusal cortex is associated with the aura period. In addition, various regions including thalamus as well as cingulate cortex, cerebellum, periaqueductal gray, hypothalamux, pons, spinal trigeminal nucleus, visual cortex, middle frontal cortex, somatosensory and temporo-occipital cortex can be related with ictal phase of the migraine [8].

In the pathogenesis of migraine, the role of the thalamus has been considered as the relay center for ascending nociceptive information, via the trigemino-vascular pain pathway, from lower brain areas to various cortical regions [9]. The presence of multisensory symptoms during migraine attacks and the central role of the thalamus indicate a potential involvement of the thalamus in the pathogenesis of migraine [9]. Recent neuroimaging research, with advantages of brain MRI techniques, has revealed an expanding spectrum of additional structural and functional roles of the thalamus in migraine, which could provide a better understanding of the pathophysiology of migraine. In a structural study, there were no changes in the overall volume of the thalamus in patients with migraine compared with healthy controls [6, 7]. However, in another study with 37 migraineurs, T1 relaxation time was significantly shorter in the thalamus of migraineurs compared with healthy controls [3]. In addition, the magnetization transfer ratio was higher and the T2* relaxation time was shorter in migraineurs with aura [3]. These data reveal broad microstructural alterations in the thalamus of migraineurs compared healthy controls, suggesting increased iron deposition and myelin content/cellularity. In a functional study, 17 patients with migraine underwent resting-state functional MRI scan during migraine attacks [4]. The authors found increased functional connectivity between the right thalamus and several contralateral brain regions, such as superior parietal lobule, insular cortex, primary motor cortex, supplementary motor area, and orbitofrontal cortex, whereas decreased functional connectivity was noted between the right thalamus and ipsilateral brain areas, including the primary somatosensory cortex and premotor cortex [4]. The study suggested that network connectivity between the thalamus and the pain-modulating as well as pain-encoding cortical areas were affected during migraine attacks [4]. Another study assessed the local levels of glutamate/glutamine and gamma-aminobutyric acid in the thalamus in patients with migraine and healthy controls using proton magnetic resonance spectroscopy [5]. That study revealed significantly increased glutamine levels in the thalamus, suggesting increased regional excitability [5]. All of these studies commonly demonstrate the pivotal role of the thalamus in the pathogenesis of migraine.

The aim of this study was to investigate the alterations of thalamic nuclei volumes and the intrinsic thalamic network in patients with migraine compared with healthy controls. We hypothesized that there would be significant alterations of thalamic nuclei volumes or network, which could be related to the pathogenesis of migraine.

## Methods

### Subjects

This was a cross-sectional study conducted in a tertiary hospital. This study was approved by our hospital’s institutional review board. We prospectively enrolled the subjects according to the following criteria: [1] patients had visited the neurology department of our hospital between August 2018 and July 2019, [2] patients were newly diagnosed with migraine without aura based on the International Classification of Headache Disorders, [10] who had no preventive medications for migraine, [3] patients had normal brain MRI on fluid-attenuated inversion recovery and T2-weighted imaging with visual inspection, and [4] patients had no history of medical, neurological, or psychiatric disease.

The control group included 40 age- and sex-matched healthy subjects. All healthy control subjects had normal neurological findings and no history of medical, neurological, or psychiatric disease, including any types of headache. All had a normal MRI on visual inspection.

### Brain MRI

All MRI scans were performed using a 3.0 T MRI scanner (AchievaTx, Phillips Healthcare, Best, The Netherlands) equipped with a 32-channel head coil. All patients were interictal state of headache at the time of MRI scan. All subjects, including patients with migraine and healthy controls, underwent contiguous three-dimensional volumetric T1-weighted imaging with a high sagittal resolution appropriate for the analysis of structural volume. The three-dimensional T1-weighted images were obtained using a turbo-field echo sequence with the following parameters: TI = 1300 ms, TR/TE = 8.6/3.96 ms, flip angle = 8°, and 1 mm^3^ isotropic voxel size.

### Analysis of thalamic nuclei volumes

**Fig. 1:**
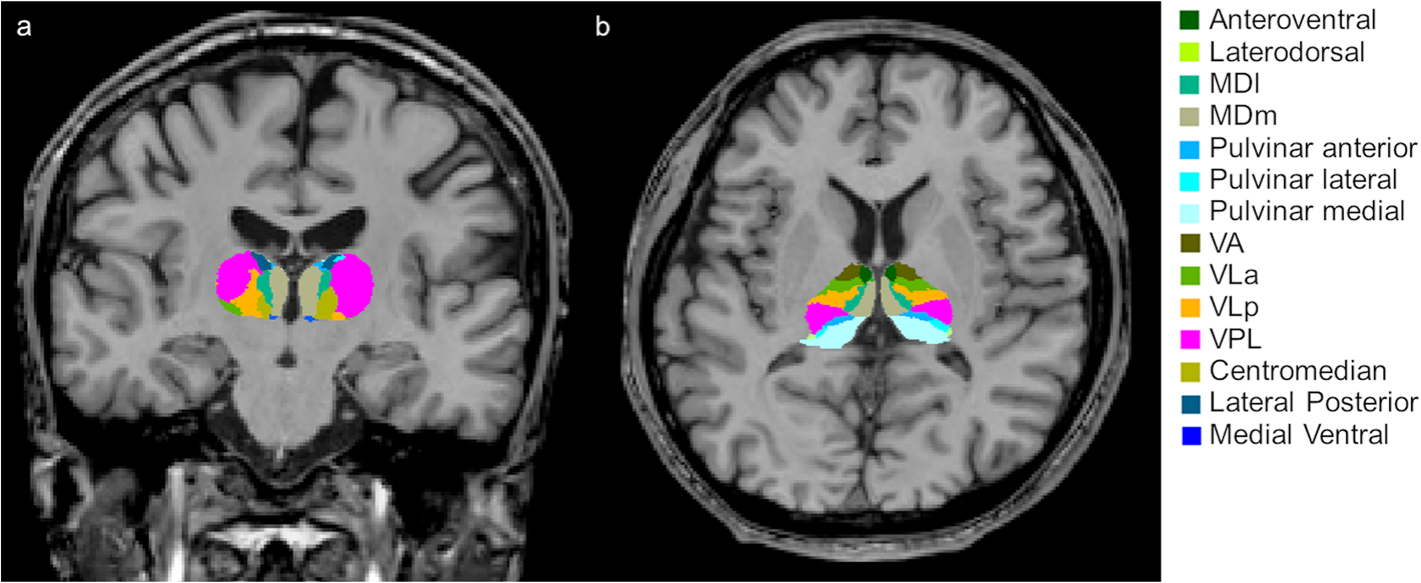
Example of segmentation of thalamic nuclei Segmentations and labels of thalamic nuclei in the coronal **a** and axial **b** plane generated by FreeSurfer (not all segmentations are shown). The segmentations are overlaid on the T1-weighted scan. MDl: mediodorsal lateral parvocellular, MDm: mediodorsal medial magnocellular, VLa: ventral lateral anterior, VLp: ventral lateral posterior, VPL: ventral posterolateral, VA: ventral anterior nucleus.

Volumetric analysis was performed using the “recon-all” function in the FreeSurfer program (http://surfer.nmr.mgh.harvard.edu/). This step included several steps of imaging processes, including normalization of signal intensity, skull stripping to separate areas of the skull in the normalized space, and automatic segmentation. Then, individual thalamic nuclei were segmented. We obtained the absolute individual thalamic nuclei volumes from these automated methods, which is a Bayesian segmentation method based on a probabilistic atlas derived from histology (Fig. 1) [11]. Next, the volumetric measures were calculated using the following equation: the structural volumes (%) = (absolute structural volumes/total intracranial volumes) × 100.

### Analysis of intrinsic thalamic network

We performed a volume-based analysis of the intrinsic thalamic network using Brain Analysis using Graph Theory (BRAPH; http://braph.org) [12]. They were built for each group as a collection of nodes representing brain regions (individual thalamic nucleus volumes) connected by edges corresponding to the connections between them. In the intrinsic thalamic network analysis, we used the volumes of 50 individual thalamic nuclei, including right and left anteroventral nuclei in the anterior group; right and left laterodorsal and lateral posterior nuclei in the lateral group; right and left ventral anterior, ventral anterior magnocellular, ventral lateral anterior, ventral lateral posterior, ventromedial, and ventral posterolateral nuclei in the ventral group; right and left central medial, central lateral, paracentral, centromedian, and parafascicular nuclei in the intralaminar group; right and left paratenial, medial ventral, mediodorsal medial magnocellular, and mediodorsal lateral parvocellular nuclei in the medial group; and right and left lateral geniculate, medial geniculate, suprageniculate, pulvinar anterior, pulvinar inferior, pulvinar lateral, and pulvinar medial nuclei in the posterior group. The edges were calculated as the partial correlation coefficients between every pair of brain regions while controlling for the effects of age and sex. For each group, a structurally weighted connectivity matrix was built. To detect differences between groups in the intrinsic thalamic network topology based on graph theoretical analysis, we calculated the average strength, characteristic path length, global efficiency, local efficiency, clustering coefficient, modularity, assortativity, and small-worldness index [12]. We investigated the alterations of these network measures in the patients with migraine compared with healthy controls.

### Statistical analysis

Comparisons of the factors were analyzed using the chi-squared test for categorical variables and the Student’s *t*-test for continuous variables. In the comparison of the network measures, we tested the statistical significance of the differences using nonparametric permutation tests with 1000 permutations. We quantified correlations between thalamic nuclei volumes and clinical variables, such as disease duration, attack frequency, headache intensity (visual analog scale) using Pearson’s correlation test. Categorical variables were presented as the frequency and percentage, whereas continuous variables were presented as the mean value ± standard deviation. A two-sided *p*-value less than 0.05 was considered to indicate statistical significance for all analysis. However, in the analysis of the volume differences in the thalamic nucleus between the two groups, we set the significant *p*-value as 0.001 (0.05/50 = 0.001, Bonferroni corrections). When it comes to intrinsic thalamic network analysis, a *p*-value of 0.006 (0.05/8 = 0.006, Bonferroni corrections) was considered as significant. In terms of correlation analysis, we set the significant *p*-value as 0.001 with Bonferroni corrections. All statistical tests were performed using MedCalc® (MedCalc Software version 19, Ostend, Belgium; https://www.medcalc.org; 2019).

## Results

### Clinical characteristics of the subjects

Table 1 shows the clinical characteristics of patients with migraine and healthy subjects. The mean age of the patients with migraine was 37.9 years. More than two-thirds of the patients with migraine were female. Patient age and male-to-female ratio were not different between the patients with migraine and healthy subjects.

**Table 1:** Clinical characteristics of the patients with migraine and healthy controls

	Patients with migraine (*n* = 35)	Healthy controls (*n* = 40)	*p*-value
Age, years	37.9 ± 10.7	35.4 ± 6.8	0.2241
Male, n (%)	9 (25.7)	9 (22.5)	0.7467
Disease duration	9.2 ± 7.8		
Attack frequency per month, n	3.8 ± 3.7		
Headache intensity, visual analog scale	6.9 ± 1.5		

### Thalamic nuclei volumes

**Fig. 2:**
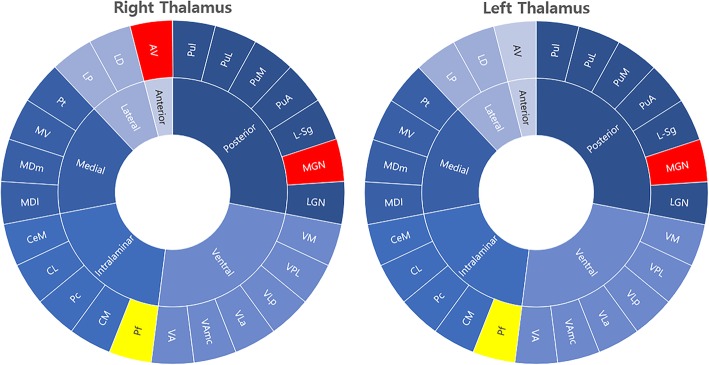
Thalamic nuclei with significant volume alterations in patients with migraine Regions in red represent increased volumes and regions in yellow reveal decreased volumes in patients with migraine as compared with healthy controls. The figure shows that the right anteroventral and right and left medial geniculate nuclei volumes are increased, whereas the right and left parafascicular nuclei volumes are decreased in patients with migraine. AV: anteroventral, LD: laterodorsal, LP: lateral posterior, VA: ventral anterior, VAmc: ventral anterior magnocellular, VLa: ventral lateral anterior, VLp: ventral lateral posterior, VPL: ventral posterolateral, VM: ventromedial, CeM: central medial, CL: central lateral, Pc: paracentral, CM: centromedian, Pf: parafascicular, Pt: paratenial, MV: medial ventral, MDm: mediodorsal medial magnocellular, MDl: mediodorsal lateral parvocellular, LGN: lateral geniculate, MGN: medial geniculate, L-Sg: suprageniculate, PuA: pulvinar anterior, PuM: pulvinar medial, PuL: pulvinar lateral, Pul: pulvinar inferior nucleus.

Table 2 reveals the volume of the thalamus as a whole and of the individual thalamic nucleus. Right and left thalamic volumes as a whole were not different between the patients with migraine and healthy controls. However, when it comes to the individual thalamic nucleus, there were significant differences between the patients with migraine and healthy controls (Fig. 2). The right anteroventral and right and left medial geniculate nuclei volumes were significantly increased (0.00985% vs. 0.00864%, *p* = 0.0002; 0.00929% vs. 0.00823%, *p* = 0.0005; 0.00939% vs. 0.00769%, *p* < 0.0001; respectively), whereas the right and left parafascicular nuclei volumes were decreased in the patients with migraine compared with healthy controls (0.00359% vs. 0.00435%, *p* < 0.0001; 0.00360% vs. 0.00438%, *p* < 0.0001; respectively), even after multiple corrections.

**Table 2:** Differences in the individual thalamic nuclei volumes between patients with migraine and healthy controls

	Patients with migraine		Healthy controls				
Thalamic nucleus	Mean, %	SD, %	Mean, %	SD, %	*p*-value		
**Lt. whole thalamus**	0.47280	0.04550	0.45970	0.06109	0.2997		
**Rt. whole thalamus**	0.45230	0.04183	0.43560	0.05335	0.1406		
**Anterior group**							
Lt. anteroventral	0.00922	0.00116	0.00834	0.00163	0.0098		
Rt. anteroventral	0.00985	0.00126	0.00864	0.00135	*0.0002		
**Lateral group**							
Lt. laterodorsal	0.00204	0.00053	0.00169	0.00050	0.0040		
Rt. laterodorsal	0.00201	0.00046	0.00165	0.00064	0.0069		
Lt. lateral posterior	0.00912	0.00119	0.00818	0.00134	0.0021		
Rt. lateral posterior	0.00874	0.00105	0.00790	0.00151	0.0076		
**Ventral group**							
Lt. ventral anterior	0.02742	0.00261	0.02588	0.00380	0.0470		
Rt. ventral anterior	0.02692	0.00236	0.02557	0.00319	0.0425		
Lt. ventral anterior magnocellular	0.00237	0.00026	0.00219	0.00034	0.0117		
Rt. ventral anterior magnocellular	0.00238	0.00023	0.00222	0.00033	0.0242		
Lt. ventral lateral anterior	0.04659	0.00513	0.04492	0.00627	0.2156		
Rt. ventral lateral anterior	0.04439	0.00403	0.04412	0.00557	0.8104		
Lt. ventral lateral posterior	0.06329	0.00748	0.06184	0.00873	0.4459		
Rt. ventral lateral posterior	0.05991	0.00595	0.05942	0.00811	0.7722		
Lt. ventral posterolateral	0.07074	0.00809	0.06968	0.01077	0.6367		
Rt. ventral posterolateral	0.06738	0.00743	0.06448	0.00992	0.1610		
Lt. ventromedial	0.00161	0.00018	0.00163	0.00026	0.6798		
Rt. ventromedial	0.00164	0.00019	0.00166	0.00028	0.7851		
**Intralaminar group**							
Lt. central medial	0.00461	0.00049	0.00433	0.00076	0.0641		
Rt. central medial	0.00474	0.00056	0.00441	0.00069	0.0306		
Lt. central lateral	0.00253	0.00057	0.00227	0.00056	0.0554		
Rt. central lateral	0.00266	0.00058	0.00231	0.00059	0.0128		
Lt. paracentral	0.00025	0.00003	0.00025	0.00005	0.8959		
Rt. paracentral	0.00025	0.00003	0.00024	0.00004	0.7118		
Lt. centromedian	0.01752	0.00187	0.01754	0.00257	0.9710		
Rt. centromedian	0.01722	0.00223	0.01743	0.00238	0.6950		
Lt. parafasicular	0.00360	0.00056	0.00438	0.00067	*< 0.0001		
Rt. parafasicular	0.00359	0.00069	0.00435	0.00057	*< 0.0001		
**Medial group**							
Lt. paratenial	0.00051	0.00006	0.00048	0.00007	0.0619		
Rt. paratenial	0.00048	0.00006	0.00045	0.00007	0.0501		
Lt. medial ventral	0.00084	0.00011	0.00076	0.00013	0.0021		
Rt. medial ventral	0.00082	0.00012	0.00072	0.00014	0.0018		
Lt. mediodorsal medial magnocellular	0.05298	0.00633	0.04999	0.00760	0.0703		
Rt. mediodorsal medial magnocellular	0.05160	0.00573	0.04793	0.00681	0.0146		
Lt. mediodorsal lateral parvocellular	0.01831	0.00241	0.01737	0.00263	0.1143		
Rt. mediodorsal lateral parvocellular	0.01827	0.00238	0.01675	0.00232	0.0066		
**Posterior group**							
Lt. lateral geniculate	0.01474	0.00213	0.01342	0.00218	0.0099		
Rt. lateral geniculate	0.01419	0.00197	0.01330	0.00151	0.0304		
Lt. medial geniculate	0.00939	0.00116	0.00769	0.00133	*< 0.0001		
Rt. medial geniculate	0.00929	0.00132	0.00823	0.00121	*0.0005		
Lt. suprageniculate	0.00095	0.00020	0.00077	0.00019	0.0001		
Rt. suprageniculate	0.00098	0.00017	0.00087	0.00019	0.0130		
Lt. pulvinar anterior	0.01548	0.00182	0.01537	0.00226	0.8224		
Rt. pulvinar anterior	0.01442	0.00155	0.01385	0.00179	0.1446		
Lt. pulvinar medial	0.07370	0.00823	0.07347	0.01033	0.9174		
Rt. pulvinar medial	0.06754	0.00801	0.06553	0.00828	0.2895		
Lt. pulvinar lateral	0.01131	0.00149	0.01280	0.00262	0.0041		
Rt. pulvinar lateral	0.01009	0.00127	0.01061	0.00173	0.1460		
Lt. pulvinar inferior	0.01369	0.00178	0.01442	0.00212	0.1103		
Rt. pulvinar inferior	0.01294	0.00187	0.01298	0.00186	0.9264		

*SD* standard deviation, *Lt* left, Rt: right

*p < 0.001

### Intrinsic thalamic network

Table 3 displays the intrinsic thalamic network in the patients with migraine and healthy controls. The network measures, such as average strength, characteristic path length, global efficiency, local efficiency, clustering coefficient, modularity, assortativity, and small-worldness index, in the patients with migraine were not different from those in the healthy controls, which suggested no alterations of the intrinsic thalamic network in the patients with migraine.

**Table 3:** Differences in the intrinsic thalamic network between the patients with migraine and healthy controls

	Patients with migraine	Healthy controls	CI lower	CI upper	Difference	*p*-value
Average strength	25.4044	29.3551	−7.5212	8.1153	3.9508	0.457
Characteristic path length	2.0688	1.8213	−0.5941	0.5895	−0.2475	0.511
Global efficiency	0.5295	0.6076	−0.1348	0.139	0.0781	0.362
Local efficiency	2.003	2.6346	−1.0874	1.0751	0.6316	0.343
Clustering coefficient	0.502	0.5827	−0.1675	0.1637	0.0806	0.481
Modularity	0.0389	0.0204	−0.0437	0.0353	−0.0185	0.493
Assortativity	−0.029	−0.0347	− 0.0417	0.0423	− 0.0057	0.819
Small-worldness index	0.9724	0.9674	−0.0345	0.0429	−0.005	0.741

*CI* 95% confidence interval

### Correlation analysis

Some measures of the thalamic nuclei volumes were correlated with clinical variables. The disease duration was negatively correlated with left medial geniculate nucleus (r = − 0.459, *p* = 0.024), and visual analog scale was also negatively correlated with right lateral geniculate, right lateral posterior, and right pulvinar inferior nucleus (r = − 0.343, *p* = 0.043; r = − 0.343, *p* = 0.043; r = − 0.434, *p* = 0.091; respectively). However, after multiple corrections, there were no significant correlations between them.

## Discussion

The main finding of this study was that anteroventral and medial geniculate nuclei volumes were significantly increased with decreased volumes of parafascicular nuclei in the patients with migraine without aura compared with healthy controls, despite the fact that thalamic volumes as a whole were not changed. However, the intrinsic thalamic network was not different between them.

Only one study had investigated thalamic nucleus volumes in the patients with migraine [13]. They found significant volume reductions in central nuclear complex, anterior nucleus, and lateral dorsal nucleus. Because the central nuclear complex belongs to the intralaminar nuclei and includes the central medical and the parafascicular nuclei, the results of the previous study was partially in agreement with our study [13]. However, the previous study had several limitations compared to our study. Their patients were a heterogeneous group. They pooled data of patients with migraine with and without aura. In addition, the patients were enrolled in the four centers, which had different scanners with large variability in coil and gradient properties between them. In addition, they segmented the thalamus into 10 thalamic nuclei, only. We investigated the alterations of 25 individual thalamic nuclei volumes, and firstly analyzed the intrinsic thalamic network in patients with migraine.

The anteroventral nucleus receives dense limbic-related projections from the mammillary nuclei via the mammillo-thalamic tract and the medial temporal lobe via the fornix [14]. The output of this nucleus is primarily directed to the cingulate gyrus through the anterior limb of the internal capsule [14]. Thus, the anteroventral nucleus is an important synaptic station in the Papez circuit, which is related to emotion and memory acquisition [14]. We found that the volume of the anteroventral nucleus was increased in patients with migraine. Migraine is related to highly specific conditioning or sensitization to pain-related stimuli [15]. Sensitization refers to the process in which neurons become increasingly responsive to nociceptive or non-nociceptive stimulation with decreased response thresholds [15]. In the patients with migraine, not only nociceptive pain but also emotional words or emotional negative affect may act as migraine triggers [16]. Emotional stress is one of the most common triggers of acute migraine attack, attributed to about 80% of attacks [17]. In addition, patients with migraine have higher levels of perceived stress than healthy controls [18]. From these results, we can speculate that the increased volume of the anteroventral nucleus may produce a sensitization to emotional stress in patients with migraine.

The medial geniculate nucleus is considered to be part of the lateral thalamic nuclear group. The medial geniculate nucleus receives ascending auditory input via the brachium of the inferior colliculus and projects to the primary auditory cortex in the temporal lobe [14]. Thus, the medial geniculate nucleus is thought to be primarily responsible for auditory perception. Patients with migraine often report an aversion to various sensory stimuli during an acute attack, such as sound (phonophobia) [19]. Phonophobia can be defined as aversion to normally nonaversive sounds. This symptoms are reported in 70% to 80% of patients with migraine during an acute attack [20]. It is a plausible explanation that increased volumes of the medial geniculate nucleus might be related to phonophobia in patients with migraine. An interesting report using positron emission tomography examined the changes in regional cerebral blood flow as an index of neuronal activity in the human brain during migraine attacks [21]. During the attacks, increased blood flow was found in the auditory association cortex [21].

The parafascicular nucleus is one of the intralaminar nuclei, which are characterized by their projections to the neostriatum and to other thalamic nuclei, along with diffuse projections to the cerebral cortex [14]. Thalamic regions have been traditionally proposed to support pain processing and arousal [22, 23]. We found that patients with migraine had significantly decreased parafasicular nucleus volumes. In an animal study on familial hemiplegic migraine (FHM) that examined the effects of an FHM-1 mutation in the central trigeminal nociceptive pathway, transgenic mice expressing the FHM-1 mutation had more activation in the intralaminar nuclei following nociceptive trigemino-vascular stimulation when compared with wild-type animals [24]. FHM is a subtype of migraine characterized by hemiplegic aura, and two-thirds of patients with FHM also experience typical migraine attacks [25]. We can assume that alterations of the parafascicular nucleus could be related to the pathogenesis of migraine attacks. Furthermore, the parafascicular nucleus projects to the rostral and lateral areas of the frontal lobe but is more closely related with frontal lobe [14]. A previous meta-analysis on changes in gray matter in patients with migraine showed that the migraineurs had decreases in gray matter volume mainly in the frontal lobe, such as the inferior frontal gyrus, precentral gyrus, and middle frontal gyrus [26]. This result is in agreement with the findings of our present study.

We also investigated the intrinsic thalamic network based on individual thalamic nuclei volumes and found no alterations of the thalamic network in the patients with migraine compared with healthy controls. Structural connectivity refers to anatomical connections linking a set of neural elements [27]. There is a lot of evidence on the abnormal thalamo-cortical network in patients with migraine using functional MRI data [28] and diffusion tensor imaging study [29]. However, we focused on the intrinsic thalamic network and demonstrated its well-preserved status in patients with migraine despite alterations in the volume of individual thalamic nuclei.

The strength our study was that we enrolled only newly diagnosed patients with migraine and included migraine without aura to increase the homogeneity of subjects group. In addition, this is the first migraine research study to investigate the various thalamic nuclei volumes and focus on alterations of the thalamic nuclei volume and network compared with healthy controls.

However, this study has several limitations. First, this study used a cross-sectional design. This design makes it difficult to discover the causal relationship between structural changes and clinical features and the role of the thalamus in the pathophysiology of the disorder. Longitudinal studies with larger sample sizes are needed to confirm our results. Second, we used only the FreeSurfer program based on the T1-weighed images for the segmentation of the thalamic nuclei. It was suggested that a multimodal imaging strategy (T1- and T2-weighted images as well as diffusion tensor images) could improve the accuracy of thalamic segmentation [30]. However, among the most sophisticated programs of MRI analysis currently available, the FreeSurfer program represents a set of automated tools most widely used to reconstruct the brain’s structures. FreeSurfer usually offers a higher and more robust reproducibility compared with other neuroimaging analysis techniques [31]. In addition, a previous study using MRI-based thalamic nuclei volume analysis like our study demonstrated a good agreement with previous histological studies and showed an excellent test-retest reliability [32]. Third, a recent systemic review of previous functional connectivity sutides in migraine showed a poor level of reproducibility and no migraine specific pattern in functional network [33]. Migraine is a heterogeneous disorder, which might cause variation in results between studies. In addition, no sample size or power calculation guidelines are available for functional connectivity studies. It would be needed to consider multicenter studies to allow for better and more reproducible studies [33]. However, we investigated the structural connectivity based on thalamic nuclei volumes, which could be a more stable method than functional connectivity study.

## Conclusion

We found significant alterations of thalamic nuclei volumes in patients with migraine without aura compared with healthy controls, especially in the anteroventral, medial geniculate, and parafascicular nuclei. These findings might contribute to the underlying pathogenesis of the migraine.

## Data Availability

All the data supporting our findings is contained within the manuscript.

## Abbreviations

FHM: familial hemiplegic migraine
MRI: brain magnetic resonance imaging
